# Commentary: insights and future directions from a CT perfusion-based study on POCD risk prediction

**DOI:** 10.1097/JS9.0000000000003334

**Published:** 2025-09-08

**Authors:** Sibo Liu, Fushun Piao, Kai Xu

**Affiliations:** a Department of Intensive Care Unit, Central Hospital of Dalian University of Technology, Dalian, China; b Department of Painology, Central Hospital of Dalian University of Technology, Dalian, China; c Department of Neurosurgery, Central Hospital of Dalian University of Technology, Dalian, China

*Dear Editor,*

We read with genuine interest the recent article by Wang *et al*^[1]^ titled “Permeability surface elevation increased postoperative cognitive decline in patients with coronary artery bypass grafting.” This well-designed cohort study addresses a critical yet often underrecognized complication of cardiac surgery – postoperative cognitive decline (POCD). By using CT perfusion-derived permeability surface (PS) values as a marker of preoperative blood–brain barrier integrity, the authors propose a novel neuroimaging biomarker for early risk stratification.

The study included 116 patients undergoing coronary artery bypass grafting and found that 20.7% developed POCD. A 0.05 ml/100 g/min increase in right frontal PS was associated with a 49% higher risk of POCD (OR = 1.334, *P* = 0.002), even after adjusting for confounders such as age, diabetes, hypertension, left ventricular ejection fraction, and cerebrovascular history.

We commend the authors for their methodological rigor, including prospective data collection, standardized anesthesia protocols, blinded image interpretation, and the use of semi-automated postprocessing techniques. To help strengthen future research in this area, we respectfully offer the following four suggestions:

First, defining POCD as a ≥20% reduction in the Montreal Cognitive Assessment (MoCA) score may introduce misclassification. MoCA is primarily a screening tool, and fixed-percentage declines may not reflect true cognitive deterioration. Several neuropsychological guidelines suggest using *Z*-score conversions or Reliable Change Indices to reduce false positives and account for baseline variability^[2]^.

Second, 84.48% of patients had preoperative MoCA scores below 26, indicating widespread baseline cognitive impairment. The potential moderating effect of baseline cognitive reserve on PS–POCD associations was not assessed. We recommend including baseline MoCA as a covariate or stratifying patients by cognitive status in future models.

Third, CT perfusion-derived PS values are partially influenced by cerebral blood flow (CBF)^[3-5]^. Intraoperative hemodynamic fluctuations common in cardiac surgery may bias PS estimates. Future studies should consider integrating CBF measurements or multi-timepoint PS–CBF monitoring to improve interpretability.

Fourth, several key imaging parameters were not disclosed. Information on contrast injection rate, timing, scanner model, software version, and Region of Interest (ROI) delineation protocol is essential for reproducibility. We encourage future studies to follow reporting standards such as STARD or QIBA and include inter-rater reliability data^[6]^.

In summary, this study adds valuable evidence to the evolving field of perioperative neuroimaging. With further methodological refinements and cross-center validation, PS-based biomarkers may hold promise for routine POCD risk screening. We appreciate the authors’ contribution and look forward to further developments in this area.

## Data Availability

All data analyzed or generated during this study are included in the article.

## References

[1] Wang W Zhang S Yu L. Preoperative blood-brain barrier disruption increased risk of postoperative cognitive decline after coronary artery bypass grafting: a prospective cohort study. Int J Surg 2025;10–1097. doi:10.1097/JS9.0000000000002902 PMC12626559 40696925

[2] Borchers F Spies CD Feinkohl I. Methodology of measuring postoperative cognitive dysfunction: a systematic review. Br J Anaesth 2021;126:1119–27. 33820655 10.1016/j.bja.2021.01.035

[3] Lin K Kazmi KS Law M Babb J Peccerelli N Pramanik BK. Measuring elevated microvascular permeability and predicting hemorrhagic transformation in acute ischemic stroke using first-pass dynamic perfusion CT imaging. AJNR Am J Neuroradiol 2007;28:1292–98. 17698530 10.3174/ajnr.A0539 PMC7977671

[4] Bivard A Kleinig T Churilov L. Permeability measures predict hemorrhagic transformation after ischemic stroke. Ann Neurol 2020;88:466–76. 32418242 10.1002/ana.25785 PMC7496077

[5] Zhang X Zhu HC Yang D. Association between cerebral blood flow changes and blood-brain barrier compromise in spontaneous intracerebral haemorrhage. Clin Radiol 2022;77:833–39. 35786315 10.1016/j.crad.2022.05.028

[6] Bossuyt PM Reitsma JB Bruns DE. STARD 2015: an updated list of essential items for reporting diagnostic accuracy studies. Bmj 2015;351:h5527. 26511519 10.1136/bmj.h5527 PMC4623764

[7] Agha RA Mathew G Rashid R. Transparency in the reporting of Artificial INtelligence – the TITAN guideline. Prem J Sci 2025;10:100082.

